# Bony Anatomy, Soft-Tissue Adaptations and Neuromuscular Risk Factors of Shoulder Injury in Handball Players: A Narrative Review

**DOI:** 10.3390/clinpract16100195

**Published:** 2026-10-20

**Authors:** Zacharoula Papadopoulou, Angelo V. Vasiliadis, Eleni Maria Vrampa, Nikiforos Galanis

**Affiliations:** 1 School of Physical Education and Sports Science, Department of Competitive Sports, Division of Team Handball, Aristotle University of Thessaloniki, 57001 Thessaloniki, Greece; 2 Department of Physical Education and Sports Science at Serres, Aristotle University of Thessaloniki, 62122 Serres, Greece; 3 1st Orthopaedic Department, George Papanikolaou General Hospital, Medical School, Aristotle University of Thessaloniki, 57001 Thessaloniki, Greece; ngalanismed@gmail.com; 4 Department of Orthopaedic Surgery, Sports Trauma Unit, St. Luke’s Hospital, 55236 Thessaloniki, Greece; 5 Department of Physical Education and Sports Science, Aristotle University of Thessaloniki, 57001 Thessaloniki, Greece

**Keywords:** handball, shoulder injury, overhead athletes, GIRD, ERG, muscle imbalance, glenohumeral biomechanics

## Abstract

Handball is a high-intensity contact sport that imposes significant mechanical stress on the shoulder joint through repetitive, high-velocity overhead throwing, predisposing athletes to a wide spectrum of shoulder injuries. This narrative review examines the factors associated with shoulder injuries in handball players, highlighting underlying structural, adaptive and functional mechanisms across male and female athletes. A literature search of PubMed/MEDLINE was conducted to synthesize current evidence on bony anatomy, soft tissue adaptations and neuromuscular characteristics in handball cohorts. Factors associated with shoulder injury or symptoms can be broadly categorized into three conceptual domains: (i) bony anatomy and structural features, (ii) soft tissue adaptations and (iii) dynamic functional and neuromuscular factors. Bony factors include reduced acromiohumeral distance, increased humeral retrotorsion as a sport-specific skeletal adaptation and variations in glenoid morphology that may influence glenohumeral stability. Soft tissue adaptations include posterior capsular tightness, anterior capsular laxity and alterations in shoulder range of motion, particularly glenohumeral internal rotation deficit (GIRD) accompanied by external rotation gain (ERG), which may influence glenohumeral kinematics and potentially contribute to shoulder symptoms or overuse conditions. Beyond these structural features, dynamic functional factors, including shoulder rotator muscle strength imbalances, impaired scapular control and training-load management, play a central role in injury development. Therefore, a comprehensive assessment encompassing anatomical correlates, soft tissue characteristics and functional/neuromuscular characteristics may support the development of targeted injury-prevention and rehabilitation strategies in handball players.

## 1. Introduction

Handball is a high-intensity contact sport that places substantial mechanical demands on the shoulder through repetitive overhead throwing, extreme arm positions and frequent player contact. Anatomically, the glenohumeral joint sacrifices structural bony stability for high mobility, with the large humeral head contacting less than one-third of the shallow glenoid fossa [1]. While this design permits the extreme range of motion needed for high-velocity throwing, stability depends heavily on passive capsulolabral restraints and active rotator cuff muscles [1,2]. In handball, repetitive throwing exposes these soft-tissue stabilizers to severe rotational torques, heightening susceptibility to fatigue and pathology [2,3]. These sport-specific demands are associated with ligamentous, tendinous, capsular and osseous shoulder conditions, making the shoulder one of the most commonly affected joints in handball players and a frequent cause of pain, reduced performance and time lost from sport [3].

During a competitive season, a handball player performs thousands of high-velocity throws. Ball characteristics and peak velocities vary across competition levels and sexes, with elite male players using heavier 400 g balls and reaching speeds of up to 130 km/h, compared with elite female players, who reach speeds of 60–80 km/h with a 325 g ball [4,5,6]. Nevertheless, the repetitive acceleration-deceleration cycles and peak rotational forces generated across all cohorts generate peak shoulder stresses reaching up to 1.5 times body weight [7], exposing the joint to considerable mechanical loading. In addition to overuse, direct contact during shooting may produce excessive anterior glenohumeral translation, increasing the risk of capsulolabral injury, anterior instability and rotator cuff pathology [3,8]. Consequently, shoulder injuries account for a substantial proportion of both acute and overuse conditions in handball, with nearly half of athletes reporting persistent shoulder problems severe enough to limit training or competition [9,10].

While handball players across all performance levels demonstrate high injury susceptibility, individual risk is shaped by a complex interplay of repetitive overhead throwing, training loads and individual anatomical and neuromuscular characteristics [3]. Sex-related variations, such as differences in baseline joint laxity, muscle strength ratios and glenoid morphology, may contribute to differences in shoulder characteristics and injury presentation between male and female players [11,12,13,14], though much of the current foundational biomechanical literature in handball has predominantly examined male or mixed-sex cohorts. Moreover, the magnitude of tissue adaptations is heavily influenced by age, skeletal maturity and cumulative exposure, with elite athletes demonstrating more pronounced sport-specific changes than recreational or amateur players [15,16].

**Figure 1:**
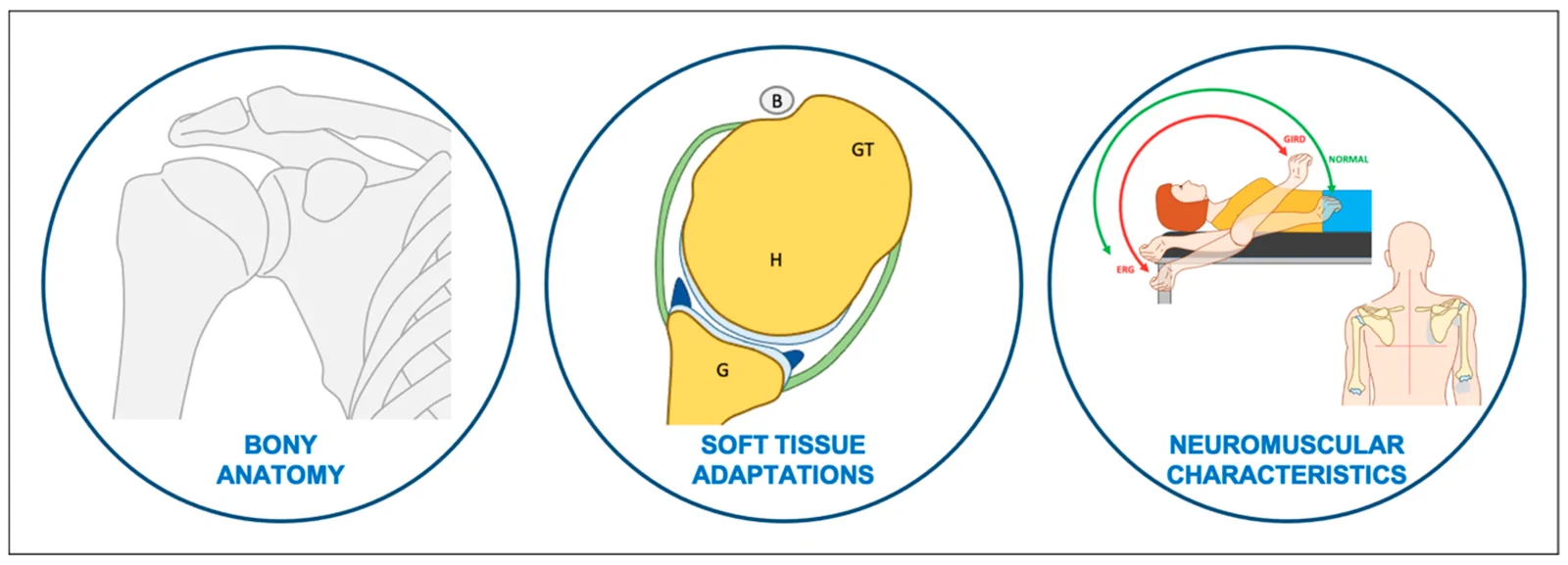
Categorization of factors associated with shoulder injury risk. Abbreviations: B, biceps tendon; GT, great tuberosity; H, humerus; G, glenoid; ERG, external rotation gain; GIRD, glenohumeral internal rotation deficit.

Accordingly, this narrative review synthesizes the current evidence regarding anatomical correlates, adaptive tissue characteristics and dynamic functional/neuromuscular factors associated with shoulder injuries in handball players (Figure 1). Particular emphasis is placed on distinguishing physiological, sport-specific adaptations to repetitive overhead throwing from maladaptive changes that increase injury susceptibility. Because these factors do not act in isolation, their clinical relevance must be considered within the broader context of cumulative shoulder loading, repetitive throwing exposure and individual physical capacity across the sport.

## 2. Methods

### 2.1. Design and Rationale

This narrative review aims to summarize existing evidence qualitatively rather than provide pooled quantitative effect estimates. Accordingly, this review was not designed as a systematic review and does not claim exhaustive coverage or full reproducibility.

### 2.2. Information Sources and Study Selection

A structured literature search was conducted using the PubMed/MEDLINE database. Search strings combined keywords and Medical Subject Headings (MeSH) terms related to handball (“handball”, “overhead athletes”), shoulder anatomy and structural adaptations (“acromiohumeral distance”, “glenoid morphology”, “humeral retrotorsion”, “GIRD”, “ERG”, “range of motion”) and injury risk (“shoulder injury”, “impingement”, “instability”, “muscle imbalance”). The search covered publications from database inception to August 2026 and was conducted on 31 August 2026. Studies were considered eligible if they investigated shoulder anatomy, structural or soft-tissue adaptations, range of motion, muscle strength, neuromuscular characteristics, training load or factors associated with shoulder symptoms or injury in handball players. Only studies published in English were considered. Studies involving male and/or female handball players of any competitive level and relevant age group were eligible. Studies unrelated to the shoulder, studies not involving handball populations and publications lacking relevant primary or review evidence were excluded. Titles and abstracts were screened for relevance, followed by full-text assessment when necessary. The reference lists of relevant studies were manually screened to identify additional studies. Particular attention was given to prospective and cross-sectional studies involving handball cohorts.

## 3. Results

Current research shows that shoulder injuries in handball players are associated with a complex interaction between bony anatomy, soft-tissue adaptations and dynamic neuromuscular factors (Table 1). Structural features, including reduced acromiohumeral distance and variations in glenoid morphology, may influence subacromial clearance and joint stability. Conversely, increased humeral retrotorsion develops during skeletal growth as a sport-specific skeletal adaptation associated with repetitive throwing. Soft-tissue characteristics commonly include posterior capsular tightness and anterior laxity, which are associated with rotational deficits, such as glenohumeral internal rotation deficits and total rotational motion losses. On a functional level, potentially modifiable factors associated with shoulder symptoms or injury include a low external-to-internal rotator strength ratio, external rotation weakness and altered scapular kinematics.

## 4. Discussion

This narrative review synthesizes current evidence on the bony, soft-tissue and neuromuscular factors influencing shoulder susceptibility in handball players. Repetitive high-velocity throwing exposes the glenohumeral joint to extreme mechanical stress and may be associated with a complex spectrum of physiological adaptations and pathological changes. By evaluating how fixed structural features interact with modifiable soft-tissue and dynamic neuromuscular parameters, this section highlights critical biomechanical targets for clinical assessment, load management and injury prevention in handball athletes.

### 4.1. Bony Anatomy and Structural Features

Bony anatomy plays a fundamental role in glenohumeral biomechanics. A reduced acromiohumeral distance, typically assessed using anteroposterior radiographs, has been associated with subacromial narrowing and rotator cuff pathology in overhead athletes [3,17]. A value of approximately < 7 mm is commonly described as a radiographic indicator of reduced acromiohumeral space [3,17]; however, this threshold should be regarded as a descriptive clinical reference rather than a validated, handball-specific predictor of shoulder injury. Rentz and Legerlotz suggested that repetitive superior migration of the humeral head during throwing, combined with insufficient subacromial clearance, may progressively increase tendon compression over time, potentially contributing to shoulder problems in handball players [17]. Inadequate shoulder strength and restricted range of motion (ROM) can exacerbate this mechanism by impairing the ability to maintain subacromial space during dynamic throwing movements [17]. In handball, where repetitive high-force overhead throws are routine, even subtle reductions in acromiohumeral distance may be clinically relevant across different competitive levels.

**Figure 2:**
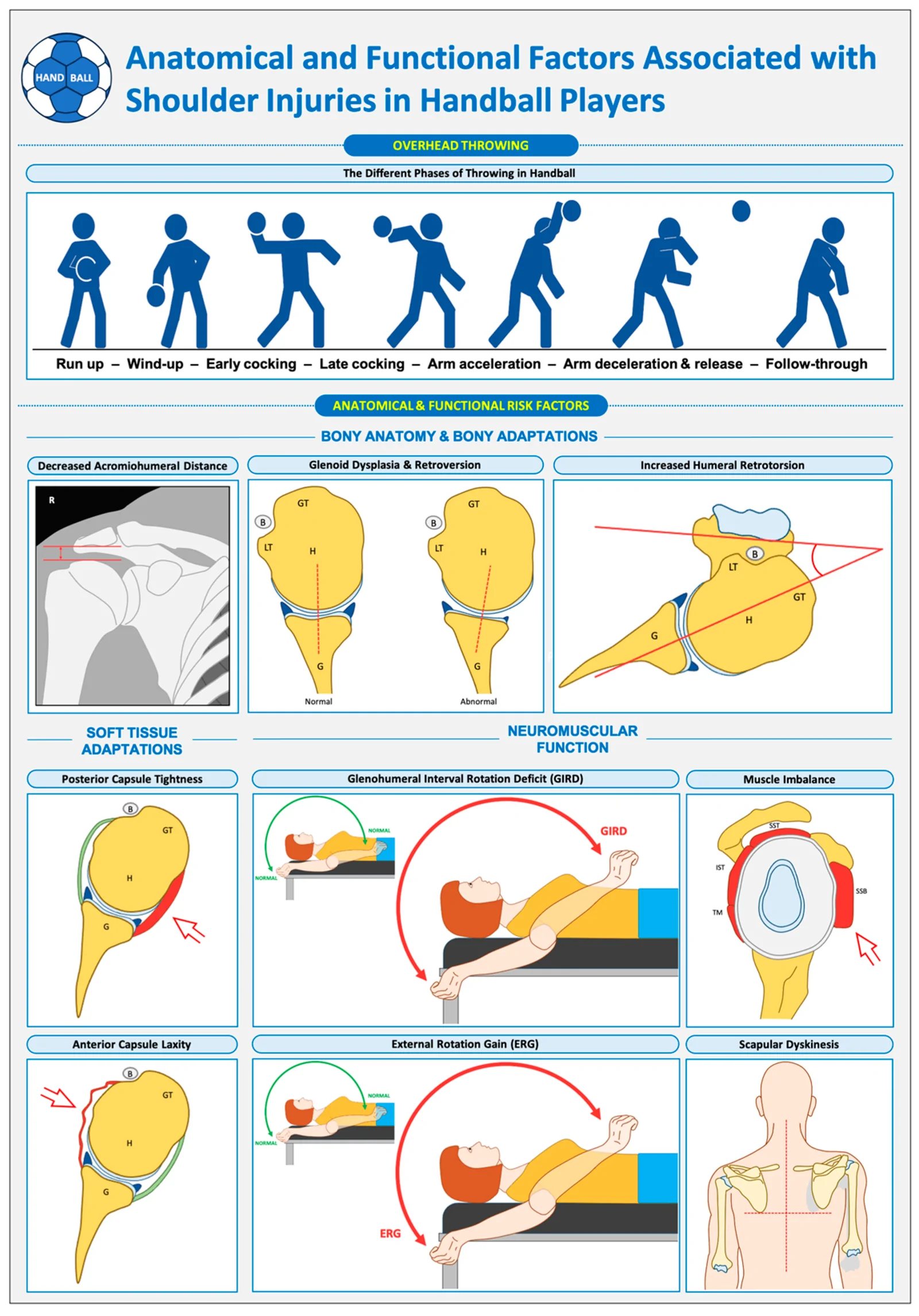
Bony anatomy and functional factors associated with shoulder injuries in handball players. Abbreviations: B, biceps tendon; GT, great tuberosity; LT, lesser tuberosity; H, humerus; G, glenoid; ERG, external rotation gain; GIRD, glenohumeral internal rotation deficit; SSB, subscapularis; SST, supraspinatus; IST, infraspinatus; TM, teres minor.

In addition, glenoid morphology plays a critical role in shoulder stability. Glenoid dysplasia, a shallow glenoid fossa, a hypoplastic scapular neck and increased glenoid retroversion may compromise the concavity–compression mechanism of the glenohumeral joint [3,12,16,25]. While these bony configurations are biomechanically plausible contributors to reduced concavity-compression stability, the current literature in handball cohorts has not demonstrated a direct, independent predictive relationship between baseline glenoid morphology and acute shoulder injury risk [12,25]. However, such structural configurations may be associated with chronic shoulder overuse conditions, although their independent contribution to injury susceptibility remains uncertain (Figure 2).

Humeral retrotorsion, defined as a posterior orientation of the humeral head relative to the distal humerus, represents another key bony adaptation to repetitive rotational loading. Overhead athletes frequently demonstrate increased humeral retrotorsion on the dominant side as an adaptive response to repetitive throwing [3]. Pieper reported greater humeral retrotorsion in the throwing arm of handball players, consistent with an adaptation associated with extensive exposure to external rotation during throwing, particularly throughout skeletal growth [16]. This aligns with theories emphasizing the effects of repetitive rotational loading on the immature skeleton, especially in athletes who begin handball participation prior to physeal closure [3,16,18]. Current evidence indicates that a side-to-side difference in humeral retrotorsion of approximately 5° to 10° is commonly observed in handball athletes as a sport-specific skeletal adaptation rather than a pathological change [15,18]. Increased humeral retrotorsion may shift the arc of rotational motion posteriorly, potentially allowing greater external rotation without increasing mechanical stress on the anterior capsulolabral structures. However, excessive retrotorsion combined with substantial GIRD, posterior capsular tightness or reduced total rotational motion may alter glenohumeral mechanics and potentially be associated with shoulder symptoms [15,18].

Although baseline humeral retrotorsion is known to vary significantly across general populations [26,27], evidence specifically examining racial or ethnic differences in humeral retrotorsion or injury resilience within handball cohorts remains limited. However, Thomas et al. demonstrated greater dominant-arm humeral retrotorsion in Latin American baseball players compared to North American counterparts [28], highlighting cohort-wide skeletal variations as an important direction for future handball research.

### 4.2. Soft Tissue Adaptations

In addition to sport-specific adaptations, individual biological traits can further influence shoulder injury susceptibility across handball athletes. In female-specific cohorts, players frequently demonstrate greater generalized ligamentous laxity, lower passive joint stiffness and distinct baseline neuromuscular control patterns, which may affect glenohumeral stability during high-velocity overhead throwing compared to male peers [29]. Furthermore, cyclic hormonal fluctuations (e.g., estrogen and relaxin) have been suggested to influence connective tissue properties specifically in female athletes, though their direct link to shoulder injury risk in female overhead athletes requires further empirical clarification [30,31]. These intrinsic features interact with repetitive throwing exposure and sport-specific adaptations, modifying overall risk profiles within specific athlete subgroups.

Soft tissue adaptations are commonly observed among handball athletes and have been associated with overuse conditions. These adaptations include posterior capsular tightness, anterior capsular laxity and rotator cuff muscle imbalance (Figure 2) [19]. Studies consistently report decreased glenohumeral internal rotation accompanied by increased external rotation in the dominant arm of handball players [18,19,25]. These changes have been attributed to repetitive tensile loading during throwing, particularly in abducted and externally rotated cocking positions, which may contribute to posterior capsular remodeling and anterior capsular attenuation [18,19,25].

Range of motion is routinely evaluated in the supine position with the shoulder abducted to 90° and the scapula stabilized [6]. Wilk et al. proposed the concept of total rotation motion (TRM), defined as the sum of passive internal and external rotation, with deficits greater than 5° in TRM between the throwing and non-throwing shoulders proposed as an indicator associated with elevated injury risk [32]. Alterations in glenohumeral ROM, specifically a loss of internal rotation termed glenohumeral internal rotation deficit (GIRD) accompanied by external rotation gain (ERG), are highly prevalent in handball players (Figure 2) [6,19]. These adaptations may reflect a combination of soft tissue constraints and underlying bony changes, such as humeral retrotorsion [6,12,18,19,25]. Myklebust et al. identified significant dominant and non-dominant ROM side-to-side differences in handball players [12], while Lubiatowski et al. demonstrated that pronounced rotational deficits, such as GIRD exceeding 20–25° and TRM deficit greater than 20°, frequently coexist with clinical shoulder pain and subacromial pathology in handball players [20], though these threshold values serve as observational markers rather than validated independent predictors of future injury. In isolation, GIRD and ERG may represent physiological adaptations to repetitive throwing; their clinical relevance may increase when internal rotation loss exceeds external rotation gain, producing a net reduction in TRM and potentially altering humeral head kinematics and mechanical loading of capsulolabral structures [6].

### 4.3. Dynamic Functional and Neuromuscular Factors

Muscle strength imbalances between the internal and external rotators of the shoulder represent a frequently observed and potentially modifiable factor associated with shoulder pain in handball players. Andrade et al. reported significantly greater internal rotator strength relative to external rotators, establishing a pronounced muscular imbalance in handball cohorts [21]. Mascarin et al. demonstrated that a resistance-band strengthening program yielded marked gains in external rotation strength, counteracting initial rotator dominance and reducing shoulder complaints in elite handball players [22]. Shoulder rotator strength is quantitatively evaluated using handheld or isokinetic dynamometry [23,33]. An external-to-internal rotation (ER/IR) strength ratio between 0.9 and 1.0 is commonly cited as a reference range; values below 0.7 or an isometric ER strength deficit exceeding 10–15% relative to the non-dominant side have been proposed as clinical benchmarks associated with increased shoulder complaints, although prospective evidence supporting these specific cut-offs remains limited [3,20,24].

These strength imbalances may influence dynamic glenohumeral stability and are associated with alterations in scapular kinematics (Figure 2) [6,21,25]. Among potentially modifiable factors, reduced external rotator strength has demonstrated a relatively consistent association with shoulder problems in prospective handball cohorts [22,34], although its isolated predictive capacity remains moderate when uncoupled from load and kinematic variables. Conversely, dynamic functional parameters, such as scapular dyskinesis must be distinguished from fixed structural traits. Scapular dyskinesis represents a dynamic movement characteristic involving altered scapular positioning during coupled glenohumeral movement [35]. Rather than an unchangeable anatomical defect, dyskinesis is a functional alteration that may result from periscapular fatigue or altered motor control strategies, particularly involving delayed activation or weakness of the serratus anterior and lower trapezius combined with pectoralis minor tightness [35,36]. Prospective data suggest that isolated scapular dyskinesis has limited value as an independent predictor of shoulder injury [3,8]; instead, it may be considered a physical manifestation associated with acute periscapular fatigue, GIRD and rotator cuff weakness [35,36,37].

Handball players presenting with reduced ROM and diminished external rotation strength have a higher prevalence of scapular dyskinesis and shoulder complaints [3,8], consistent with an association between muscle weakness and altered scapulothoracic control. Furthermore, recent evidence suggests that shoulder problems may be influenced by the dynamic interaction between physical capacity and accumulated workload. Handball players with greater trunk rotation power, external rotation flexibility and lower-body strength may still experience shoulder problems if shoulder loading increases rapidly throughout a competitive season, with internal load measures showing associations with shoulder symptoms [38].

Standard anthropometric measures, such as absolute body mass and body mass index (BMI) have not demonstrated direct, independent associations with overuse shoulder injury risk in handball cohorts [10,39]. Instead, evidence highlights the clinical importance of muscle strength normalized to body weight (e.g., isometric external rotation force per kilogram) and proportional muscle balance ratios (e.g., ER/IR ratios) [24]. Injury susceptibility may reflect interactions between normalized strength capacity, kinetic chain efficacy and cumulative throwing demands, rather than absolute body weight alone.

### 4.4. Practical Recommendations for Injury Prevention

Practical injury prevention strategies in handball may integrate pre-season screening, individualized physical conditioning and structured workload management. Handball-specific intervention studies support the use of programs targeting external rotator strength and shoulder function, while recommendations concerning posterior shoulder flexibility, trunk and lower-extremity strength, kinetic-chain efficiency and progressive throwing exposure are also informed by broader overhead-athlete and shoulder rehabilitation literature [40,41]. Secondary prevention approaches in handball may include targeted interventions addressing individual deficits in ROM, rotator cuff strength and dynamic movement quality [42]. Sensorimotor retraining, closed-chain stabilization exercises and progressive eccentric rotator cuff strengthening may be incorporated to support dynamic shoulder stability and tendon load tolerance, although evidence for these specific interventions in handball-specific cohorts remains limited [43]. Eccentric loading has been proposed to support tendon adaptation under mechanical stress, whereas balanced concentric-eccentric strengthening may support overall shoulder capacity in asymptomatic handball athletes [42,43].

While baseline pre-season screening is widely advocated, its ability to predict individual injury risk remains limited due to modest predictive accuracy and high false-positive rates. Consequently, screening should not be used as an isolated injury-prevention tool or an accurate predictor of future pathology [44]. Instead, periodic assessment of shoulder ROM, external rotator strength, scapular control and throwing workload should form a continuous surveillance framework to track longitudinal adaptations, detect maladaptive changes early and guide individualized preventive interventions [42,44]. Monitoring both functional performance and internal training load within an ongoing surveillance model may help guide physical adaptation and may contribute to reducing the incidence and severity of shoulder disorders in handball players.

## 5. Limitations

Several methodological limitations of this narrative review warrant consideration when interpreting the synthesized findings. First, limiting the primary literature search to the PubMed/MEDLINE database may have omitted relevant studies indexed exclusively in other databases; however, PubMed/MEDLINE provides broad coverage of the peer-reviewed biomedical, biomechanical and sports medicine literature relevant to this topic, allowing identification of a substantial proportion of the core evidence. Second, because this review was designed as a narrative synthesis rather than a systematic review, study selection and qualitative interpretation lack quantitative effect pooling and formal risk-of-bias appraisal, introducing inherent selection bias; nevertheless, this qualitative narrative design permitted a broader, clinically integrated synthesis of complex anatomical, soft-tissue and neuromuscular interactions that rigid systematic inclusion criteria might otherwise exclude. Finally, substantial cohort heterogeneity across the included literature, encompassing variations in athlete sex, age, competitive playing level and cumulative throwing exposure, limits the direct generalization of specific structural thresholds or neuromuscular cutoff values across all handball populations; at the same time, this diversity captures the true real-world spectrum of handball cohorts across different stages of athletic development.

## 6. Conclusions

Handball exposes the shoulder joint to substantial mechanical loading through high-velocity overhead throwing, extreme rotational positions and frequent physical contact, which may contribute to acute and overuse shoulder injuries. Current evidence indicates that a combination of bony anatomical features (acromiohumeral distance, humeral retrotorsion and glenoid morphology), soft tissue adaptations (capsular tightness/laxity, GIRD and ERG) and dynamic functional/neuromuscular characteristics (rotator cuff muscle strength imbalances and impaired motor control) are associated with differences in glenohumeral biomechanics and may contribute to shoulder injury susceptibility. However, these factors must be interpreted within the broader context of cumulative throwing workload and kinetic-chain mechanics, as many structural and adaptive traits represent physiological, sport-specific responses rather than inherently pathological conditions.

An integrated assessment encompassing anatomical correlates, soft tissue characteristics and functional factors, alongside individualized load management, may help identify factors associated with shoulder injury and inform targeted prevention strategies in handball players. Because high-quality, female-specific primary data remain limited compared with those from established male and mixed-sex cohorts, further prospective studies evaluating female handball players are needed to establish sex-specific baseline norms, elucidate unique biomechanical pathways and refine targeted injury-prevention protocols across all competitive levels.

## Figures and Tables

**Table 1:** Characteristics of the included studies.

Study	Design	Sample Size (*n*)	Sex (*n*)	Age (Years)	Training Profile (Years)	Sport	Factor	Findings
Almeida et al. [6]	CSCaC	64	M (36) F (28)	18–37	≥2	Professional HP	ROM	-HP with shoulder pain exhibited less IR and more ER in the throwing arm and greater GIRD and ERG than HP without shoulder pain
Myklebust et al. [12]	P	179	F (179)	22.0 ± 4.0	3.9 ± 6.6	Elite HP	ROM	-Increased ER (3.3°) and decreased IR (3.9°) between dominant and non-dominant hand
Pieper [16]	P	51	M (51)	27.0 ± 5.0	≥5	Professional HP	HR	-HR increase can be explained as an adaptation to extensive external rotation during growth
Rentz and Legerlotz [17]	CSCoC	20	M (10) F (10)	24.1 ± 4.3	12.7 ± 4.5	Experienced HP	AHD	-Reduction in AHD which is enhanced with shoulder abduction
Achenbach et al. [18]	PCS	258	M (135) F (123)	14.0 ± 0.8	NR	Elite HP	HR ROM	-Increased HR is not associated with increased risk of shoulder injury -Increased ER (9°) and decreased IN (8°) between dominant and non-dominant hand -Increased total ROM (3°) -GIRD > 7.5° found to be a borderline risk factor for overuse injuries
Schmalzl et al. [19]	ECS	67	M (67)	25.0 ± 6.0	17.0 ± 5.0	Professional HP	ROM	-GIRD is common in HP and more commonly affected than VP -GIRD > 10° and HP > 5 years of experience presents higher prevalence of PSI
Lubiatowski et al. [20]	CSCG	87	M (87)	25.0 ± 4.7	NR	Elite HP	ROM	-Higher range of IR, ER and TAM -GIRD > 20–25° co-existed with shoulder pain and PSI and ERG co-existed with RC tears
Andrade et al. [21]	CSO	27	F (27)	23.0 ± 3.4	NR	Elite HP	Strength	-IR and ER muscle strength was stronger in dominant arm (concentric contraction) due to regular training
Mascarin et al. [22]	RP	25	F (25)	≅15	NR	Elite HP	Strength	-Shoulder strength imbalance -Weakness in glenohumeral ER strength -Low ER:IR (<0.66) ratio is identified
Cools et al. [23]	CSO	64	M (32) F (32)	18–50	NR	Professional HP	Strength	-Strength differences were in favour of the dominant arm -HP had higher eccentric and isometric ER strength than VP -ER/IR ratio was lower in male HP compared to female HP reflecting an increased risk of injury
Achenbach et al. [24]	P	138	M (70) F (68)	14.1 ± 0.8	8.2 ± 2.2	Elite HP	ROM Strength	-Increased ER (8°) and decreased IR (4°) between dominant and non-dominant hand -Total ROM (4°) was greater between dominant and non-dominant hand -Weakness in glenohumeral ER strength is considered as a risk factor for overuse injury -Low ER:IR ratio is identified as risk factor for high susceptibility

Abbreviations: CSCaC, cross-sectional case–control study; P, prospective study; CSCoC, cross-sectional comparative-control study; PCS, prospective cross-sectional study; ECS, epidemiological cross-sectional study; CSCG, cross-sectional control group study; CSO, cross-sectional observational study; RP, randomized prospective study; M, male; F, female; HP, handball players; VP, volleyball players; HR, humeral retrotorsion; AHD, acromiohumeral distance; NR, not reported; ROM, range of motion; IR, internal rotation; ER, external rotation; GIRD, glenohumernal internal rotation deficit; ERG, external rotation gain; PSI, postero-superior impingement; TAM, total arch of motion; RC, rotator cuff.

## Data Availability

No new data were created or analyzed in this narrative review. Data sharing is not applicable to this article.
